# Feasibility of postural lung recruitment maneuver in children: a randomized, controlled study

**DOI:** 10.1186/s13089-020-00181-8

**Published:** 2020-07-14

**Authors:** Cecilia M. Acosta, Giovanni Volpicelli, Nadia Rudzik, Nicolás Venturin, Sebastián Gerez, Lila Ricci, Marcela Natal, Gerardo Tusman

**Affiliations:** 1 Department of Anesthesiology, Hospital Privado de Comunidad, Córdoba 4545, 7600 Mar del Plata, Buenos Aires Argentina; 2 Department of Emergency Medicine, San Luigi Gonzaga University Hospital, Orbassano, Torino Italy; 3 Department of Mathematics, Facultad de Ciencias Exactas, Universidad Nacional de Mar del Plata, Mar del Plata, Argentina

**Keywords:** Atelectasis, Body position, Lung ultrasound, Children, Lung recruitment maneuver

## Abstract

**Background:**

Pulmonary atelectasis in anesthetized children is easily reverted by lung recruitment maneuvers. However, the high airways pressure reached during the maneuver could negatively affect hemodynamics. The aim of this study is to assess the effect and feasibility of a postural lung recruitment maneuver (P-RM); i.e., a new maneuver that opens up the atelectatic lung areas based on changing the child’s body position under constant ventilation with moderated driving pressure (12 cmH_2_O) and of positive end-expiratory pressure (PEEP, 10 cmH_2_O). Forty ASA I–II children, aged 6 months to 7 years, subjected to general anesthesia were studied. Patients were ventilated with volume control mode using standard settings with 5 cmH_2_O of PEEP. They were randomized into two groups: (1) control group (C group, *n* = 20)—ventilation was turned to pressure control ventilation using a fixed driving pressure of 12 cmH_2_O. PEEP was increased from 5 to 10 cmH_2_O during 3 min maintaining the supine position. (2) P-RM group (*n* = 20)—patients received the same increase in driving pressure and PEEP, but they were placed, respectively, in the left lateral position, in the right lateral position (90 s each), and back again into the supine position after 3 min. Then, ventilation returned to baseline settings in volume control mode. Lung ultrasound-derived aeration score and respiratory compliance were assessed before (T1) and after (T2) 10 cmH_2_O of PEEP was applied.

**Results:**

At baseline ventilation (T1), both groups showed similar aeration score (P-RM group 9.9 ± 1.9 vs C group 10.4 ± 1.9; *p* = 0.463) and respiratory compliance (P-RM group 15 ± 6 vs C group 14 ± 6 mL/cmH_2_O; p = 0.517). At T2, the aeration score decreased in the P-RM group (1.5 ± 1.6 vs 9.9 ± 2.1; *p* < 0.001), but remained without changes in the C group (9.9 ± 2.1; *p* = 0.221). Compliance was higher in the P-RM group (18 ± 6 mL/cmH_2_O) when compared with the C group (14 ± 5 mL/cmH_2_O; *p* = 0.001).

**Conclusion:**

Lung aeration and compliance improved only in the group in which a posture change strategy was applied.

## Background

Anesthesia-induced atelectasis is a well-known condition in pediatric patients that is related to perioperative episodes of hypoxemia [1, 2]. The incidence of atelectasis is high and commonly appears in the most dependent lung zones where the trans-pulmonary pressure (*P*_L_ = airways pressure − pleural pressure) is the lowest [3, 4]. Mechanical ventilation using standard levels of 5 cmH_2_O of positive end-expiratory pressure (PEEP) is generally insufficient to reopen those dependent atelectasis in supine pediatric patients [3, 5]. Contrarily, a brief increase in airways pressures with a lung recruitment maneuver (RM) easily revert atelectasis because the opening pressure in these dorsal pulmonary areas is overcome [2, 6].

Many studies in healthy and sick children showed that a brief increase in plateau pressure (Pplat) and PEEP during RM is safe [6–8]. However, there are still concerns about the hemodynamic response and the mechanic stress and strain on the lung tissue caused by the maneuver in this population. In order to avoid these concerns, we have described a postural recruitment maneuver (P-RM), i.e., a ventilatory strategy aimed to obtain a lung recruitment effect by changes in body position under constant driving pressure at a moderate level of PEEP [9].

**Fig. 1:**
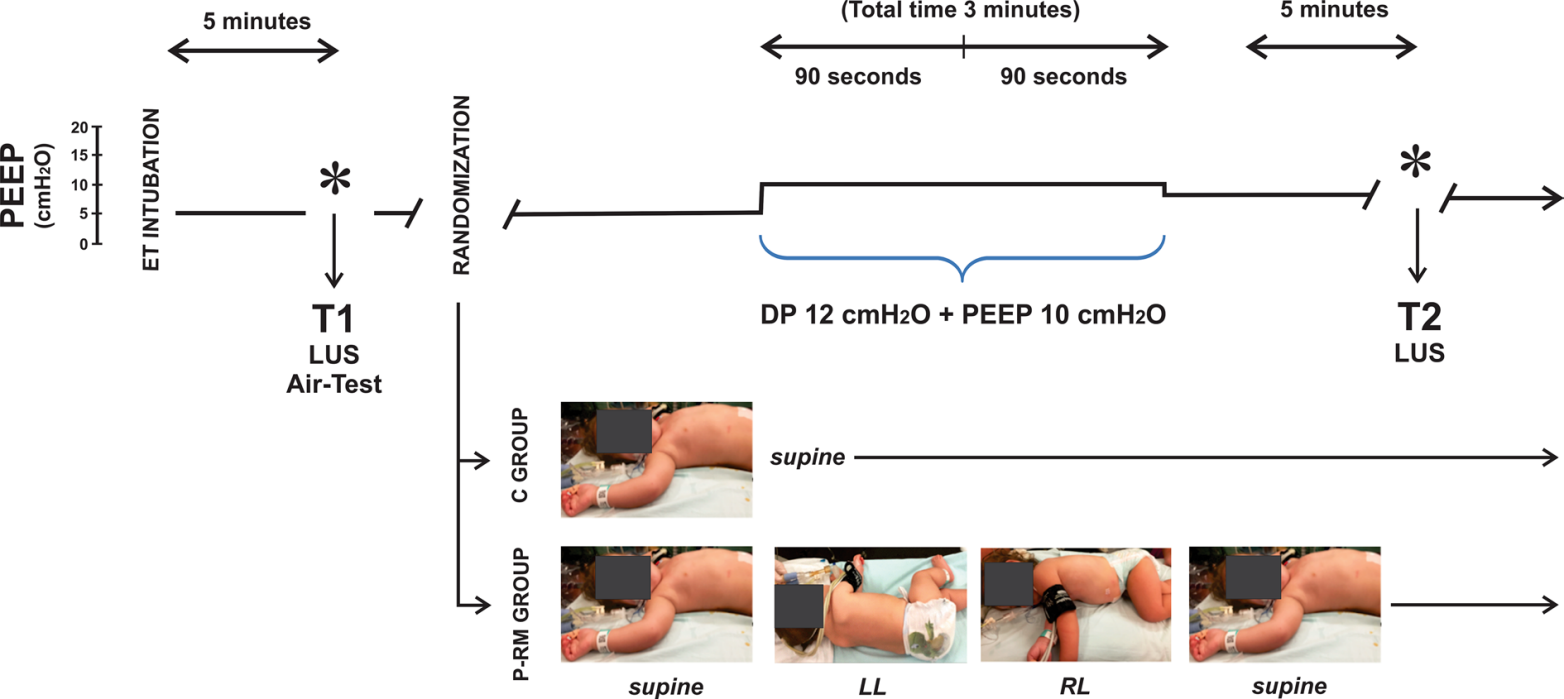
Diagram of the protocol. Ventilation was switched to pressure control mode using 12 cmH_2_O of driving pressure (DP). PEEP was increased from 5 to 10 cmH_2_O during 3 min. The control group (C group) remained supine along the protocol while children in the postural recruitment group (P-RM group) were turned to the left lateral position (LL) during 90 s and then to the right lateral (RL) for another 90 s, to finally reach the supine position again. *T1* analysis 5 min after anesthesia induction, *T2* analysis 5 min after treatment, *LUS* lung ultrasound images

The P-RM is based on the known gravitational effect on *P*_L_. Two principles explain its rationale [9]: one postulates that dorsal atelectasis can be recruited by placing this lung area in the uppermost position, which increases the local *P*_L_. The other principle follows the Laplace’s Law, which indicates that, once recruited; a ventral lung area will maintain patency when enough PEEP is applied. Therefore, the proposed P-RM consists to move the patient sequentially in: (1) the left lateral position to recruit atelectasis of the upper right lung; (2) the right lateral position to recruit the left lung atelectasis areas, while keeping open the right lung by applying enough PEEP; and (3) finally back to supine position (Fig. 1). Our preliminary data showed that P-RM using 10 cmH_2_O of PEEP and 22 cmH_2_O of Pplat was enough to open up atelectasis during anesthesia in children [9].

We hypothesized that the P-RM can re-aerate lung collapse without the need of reaching the high airways pressures obtained in standard RM. The objective of this study was to study the effect and feasibility of P-RM in anesthetized children. Main end-points of the study were lung aeration and respiratory mechanics. The primary outcome was to compare lung aeration assessed by lung ultrasound exams (LUS) between groups. The secondary outcome was to compare respiratory mechanics determined by airways resistance and dynamic respiratory compliance between groups.

## Methods

This randomized and controlled trial was performed in the operating theater of a Community Hospital. Ethical approval for this study (IRB #2919/1457/2017) was provided by the Ethical Committee of the Hospital Privado de Comunidad, Mar del Plata, Argentina (ClinicalTrials.gov NCT03141515). Written informed consent was obtained from parents of all subjects participating in the trial. The study started 20 April 2017 and ended 5 January 2018.

### Patient’s eligibility criteria

We sequentially recruited patients aged 6 months to 5 years undergoing programmed surgeries. Conditions for enrollment were: need for general anesthesia and mechanical ventilator support, American Physical Status Classification (ASA) I–II and baseline pulse oximetry saturation (SpO_2_) while breathing room air ≥ 97%. We excluded patients undergoing emergency and thoracic surgeries and patients with pre-existing pulmonary, cardiac or chest wall diseases. After this first selection, we then excluded those patients without LUS evidence of atelectasis after anesthesia induction.

### Anesthesia, ventilatory treatment and monitoring

Anesthesia was induced with sevoflurane using a circular system of the GE Aespire workstation (GE Healthcare, Madison, WI, US). Boluses of fentanyl 2 μg kg^−1^ and vecuronium 0.1 mg kg^−1^ were added before tracheal intubation with a cuffed endotracheal tube. Anesthesia was maintained with sevoflurane 0.7 minimum alveolar concentration and remifentanyl 0.3–0.5 μg kg^−1^ min. The lungs were ventilated with a volume control mode using a tidal volume (VT) of 6 mL kg^−1^, respiratory rate between 20 and 25 bpm, inspiratory-to-expiratory ratio of 1:1.5, 10% of inspiratory pause, PEEP of 5 cmH_2_O and a FIO_2_ of 0.5.

Standard EKG, non-invasive mean systemic arterial pressure (MAP), capnography, pulse oximetry and respiratory mechanics were monitored with the S5 device (GE Healthcare/Datex-Ohmeda, Helsinki, Finland). Respiratory flow and pressure signals were obtained by a pediatric mainstream gadget placed at the airways opening, from which peak airways pressure (Pip), dynamic respiratory compliance (Cdyn) and respiratory airways resistance (Rrs) were obtained.

### Gas exchange evaluated by the Air-test

Arterial oxygenation was evaluated after anesthesia induction with the *Air*-*test* using a pediatric pulse oximeter placed at the thumb (MightySat Rx, Masimo Corporation, Irvine, CA, USA) and decreasing FIO_2_ from 0.5 to 0.21 during 5 min [10, 11]. Reference SpO_2_ values breathing air in healthy patients are ≥ 97% and correspond to the anatomical shunt (~ 5–8% of the cardiac output). Any value below 97% is a marker of an additional shunt, presumably due to atelectasis, in those patients who presented baseline SpO_2_ values ≥ 97% breathing air before anesthesia induction [12].

### Lung ultrasound

LUS was performed with the ultrasound MyLab Gamma device (Esaote, Genova, Italy) using a high-frequency linear probe of 6–12 MHz. Each hemithorax was segmented into six regions using the longitudinal parasternal, anterior and posterior axillary lines and two axial lines, one above the diaphragm and the other 1 cm above the nipples [13]. The ultrasound probe was placed perpendicular to the ribs looking for the standard LUS view, where the pleura and the ventilated lung are visualized between two adjacent ribs (the *bat sign*) [13]. The probe was placed in the oblique position (along the intercostal spaces between ribs) in the areas where the typical atelectatic consolidations were detected. In general, the posterior zones of the lungs are those with the highest incidence of anesthesia-induced atelectasis [3, 4].

A LUS imaging based aeration score was calculated as previously described for children [6]. Briefly, this score is based on four LUS patterns [13–15] investigated in each of the 12 scanned thoracic areas:

1. Normal aeration (*N*): presence of the respiratory movement of the lung image relative to the chest wall (*lung sliding*) and the horizontal artifacts generated by repetition of the linear image of the pleura at regular intervals (*A lines*), with absence of sub-pleural ultrasound parenchymal signs (*B*-lines or consolidations).
2. Moderate loss of lung aeration (*B*1): presence of vertical dynamic lines, originating from the pleural line or from small sub-pleural consolidations, reaching the lowest edge of the screen (*B-lines*).
3. Severe loss of lung aeration (*B*2): multiple coalescent *B*-lines giving the aspect of a “white lung”, when the *B*-lines are so intense and numerous to occupy the whole image.
4. Complete loss of aeration (*C*): atelectasis, defined as localized sonographic consolidation, i.e., sub-pleural images with a tissue-like or hypoechoic pattern. Air bronchograms may be observed as bright echogenic branching structures within the consolidated area.

For a given thoracic area, points were allocated to the worst LUS pattern observed: *N* = 0, *B*1 = 1, *B*2 = 2 and *C* = 3. The LUS aeration score was calculated by the sum of points obtained in all the 12 lung areas, thus ranging from 0 to 36. Progressive increase of the score corresponds to loss of lung aeration.

### Protocol

After tracheal intubation atelectasis areas were diagnosed by LUS examination, consequences on arterial oxygenation were assessed by performing the Air-test (Fig. 1, T1). Patients without LUS evidence of atelectasis were excluded. Patients with atelectasis were randomized into two groups using a computerized randomization table (StatsDirect v 2.7.2; Altrincham, Cheshire, United Kingdom) by an independent and blinded operator:

- *Control group* (C group, *n* = 20). Ventilation was turned to pressure control ventilation using a fixed driving pressure of 12 cmH_2_O. PEEP was increased from 5 to 10 cmH_2_O along 3 min maintaining the supine position during the whole protocol time.
- *Postural*-*recruitment maneuver group* (P-RM group, *n* = 20). Ventilation was turned to pressure control ventilation using a driving pressure of 12 cmH_2_O and PEEP of 10 cmH_2_O, but they were immediately and sequentially placed: (1) in the left lateral position (90 s), (2) in the right lateral position (other 90 s), (3) back to the supine position (Fig. 1).

After 3′ maneuver, both groups returned to baseline ventilation adding 8 cmH_2_O of PEEP to maintain eventually the recruitment effect. Five minutes later patients were evaluated by LUS at T2 (Fig. 1). The same investigator non-blinded to treatment groups repeated LUS at each step. Respiratory data and hemodynamic parameters were collected at each protocol step.

### Statistical analysis

The null hypothesis was that lung aeration score would be similar between groups. Considering a beta-power of 80% and an alpha-error of 5% the statistical power to reject this hypothesis was calculated assuming that atelectasis would be present in 90% of patients in the C group and in only 45% of patients in the RM group [6]. A sample size of 20 patients per group was estimated. Univariate comparisons were performed between and within groups applying the Student’s *t* test. Multiple linear mixed models were adjusted to explain changes in LUS, respiratory and hemodynamic variables related to six predictive factors: age, gender, weight, surgery duration, Air-test and treatment group (fixed effects). The main factor to be analyzed was the proposed treatment.

Data are presented as *n* (%) for proportions and mean ± SD or median for continuous variables. A *p*-value < 0.05 was considered statistically significant. All calculations were performed using the R statistical package (R Core Team, 2015, Foundation for Statistical Computing, Vienna, Austria).

## Results

**Fig. 2:**
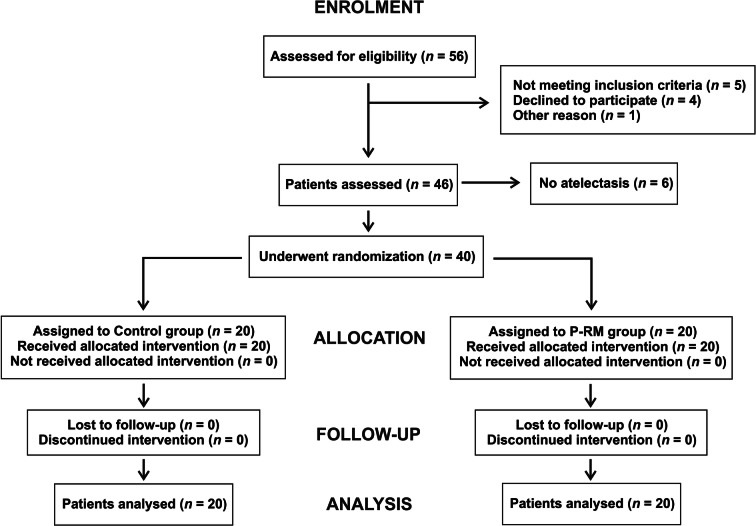
The CONSORT flow diagram

Out of the 46 examined patients, six (13%) did not show atelectasis after anesthesia induction and were excluded from the analysis. Forty patients were successfully randomized as observed in the flowchart (Fig. 2). Table 1 shows patient’s general characteristics without significant differences between groups.

**Table 1:** Patient’s data

Parameter	C-group, *n* = 20	PR-group, *n* = 20	*p* value*
Age (months)	38.8 ± 19.1	40.9 ± 16.9	0.708
Male	15 (75)	12 (60)	0.499
Female	5 (25)	8 (40)	
Weight (kg)	15.0 ± 3.9	14.8 ± 3.1	0.891
ASA I	20 (100)	20 (100)	1.00
Duration of anesthesia (min)	59.2 ± 17.9	60.9 ± 18.0	0.767
Type of surgery *n* (%)			
Laparoscopic herniorrhaphy	5 (25)	8 (40)	
Tonsillectomy	7 (35)	8 (40)	
Urological surgery	7 (35)	3 (15)	
Wrist osteosynthesis	0 (0)	1 (5)	
Resection angioma	1 (5)	0 (0)	

* Student’s t test

**Fig. 3:**
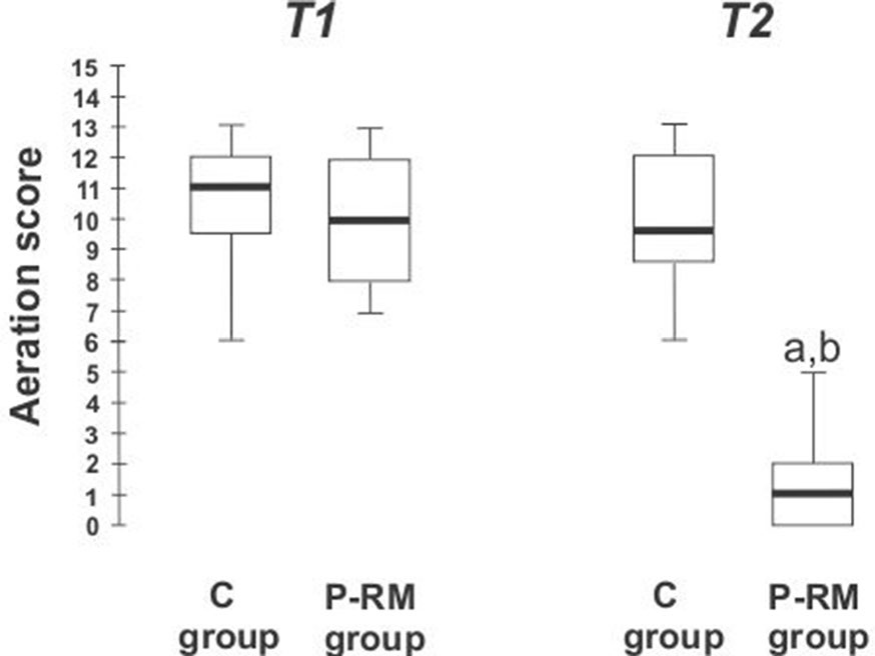
The aeration score during the study. Box-plot showing the aeration score after anesthesia induction (T1) and after 3 min of 10 cmH_2_O of PEEP in the control (C group) and postural recruitment maneuver (P-RM group) groups. Inter-group comparison, Student’s *t* test: **a** C group vs P-RM group at T2, *p* < 0.0001. **b** P-RM group T1 vs T2, *p* < 0.0001

After anesthesia induction (T1), both groups showed similar aeration score (P-RM group 9.9 ± 1.9 vs C group 10.4 ± 1.9; *p* = 0.463) and respiratory compliance (P-RM group 15 ± 6 vs C group 14 ± 6 mL/cmH_2_O; *p* = 0.517) (Fig. 3). The Air-test was positive in 19 patients of P-RM group (SpO_2_ of 93.7 ± 2.0%) and in 18 patients of the control group (SpO_2_ of 92.5 ± 2.3%; *p* = 0.248). At T2 after the 10-PEEP maneuver, the aeration score decreased in the P-RM group (1.5 ± 1.6 vs 9.9 ± 2.1; *p* < 0.001), but remained without changes in the C group (9.9 ± 2.1; *p* = 0.221). Compliance was higher in the P-RM group (18 ± 6 mL/cmH_2_O) compared with the C group (14 ± 5 mL/cmH_2_O; *p* = 0.001—Table 2).

**Table 2:** Hemodynamics and respiratory variables

Parameter	C group			P-RM group			*p* value-between groups at T2
	T1	*p* value Intra-group	T2	T1	*p* value Intra-group	T2	
Heart rate (bpm)	102 ± 15	0.033	99 ± 14	95 ± 14	0.928	97 ± 14	0.678
MAP (mmHg)	58 ± 7	0.338	58 ± 6	58 ± 6	0.763	59 ± 6	0.597
SpO_2_ (%)	98.5 ± 0.8	0.500	98.4 ± 1.1	98.8 ± 0.7	0.996	99.3 ± 0.5	0.002
PETCO_2_ (mmHg)	45 ± 3	0.006	42 ± 4	47 ± 4	< 0.0001	39 ± 3	0.005
Pip (cmH_2_O)	15 ± 2	0.002	17 ± 1	14 ± 2	0.003	16 ± 2	0.855
PEEP (cmH_2_O)	5	–	8	5	–	8	–
Cdyn (cmH_2_O)	14 ± 6	0.579	14 ± 5	15 ± 6	0.001	18 ± 6	0.0002
Rrs (cmH_2_O/L^−1^)	33 ± 8	0.941	36 ± 11	33 ± 10	0.833	35 ± 12	0.839

*T1* after anesthesia induction, *T2* after 10 cmH_2_O of PEEP in the control group (C group) and in the postural recruitment maneuver group (P-RM group), *MAP* mean arterial blood pressure, *SpO*_*2*_ pulse oximetry hemoglobin saturation, *PETCO*_*2*_ end-tidal partial pressure of carbon dioxide, *Pip* peak inspiratory pressure, *PEEP* positive end-expiratory pressure, *Cdyn* dynamic respiratory compliance, *Rrs* respiratory airways resistance. Intra-group comparison at T1 = all *p* ns

**Fig. 4:**
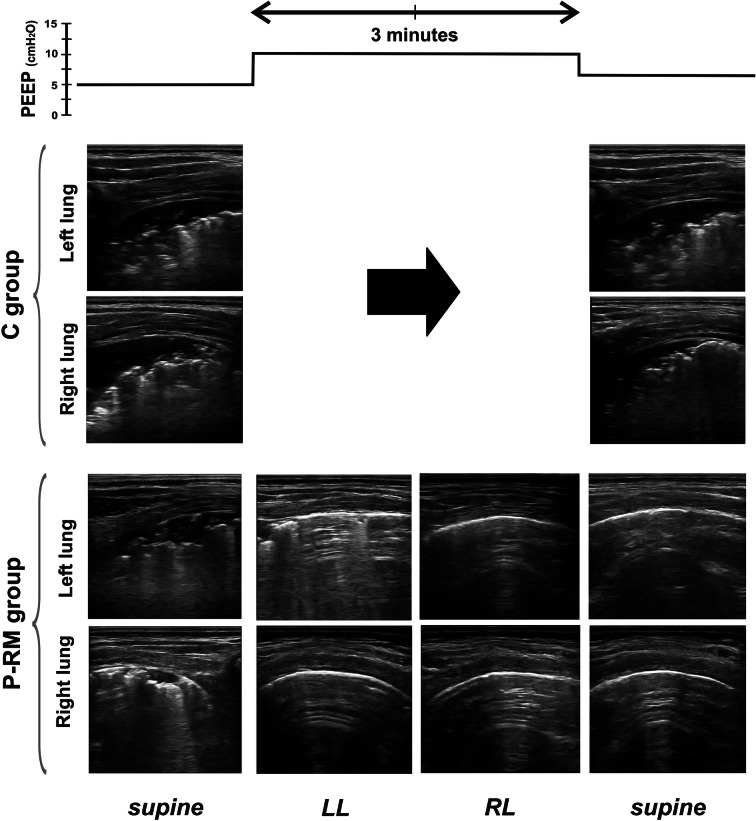
Example of the protocol in one representative patient per group. LUS images were assessed in the posterior areas in supine (dorsal lung) and in the uppermost areas in the lateral positions (ventral lung). *C group* control patients, *P-RM* postural recruitment patients, *LL* left lateral, *RL* right lateral position. Note typical atelectasis with air bronchograms before the 10-PEEP maneuver in both groups and how only the P-RM resolved it

**Fig. 5:**
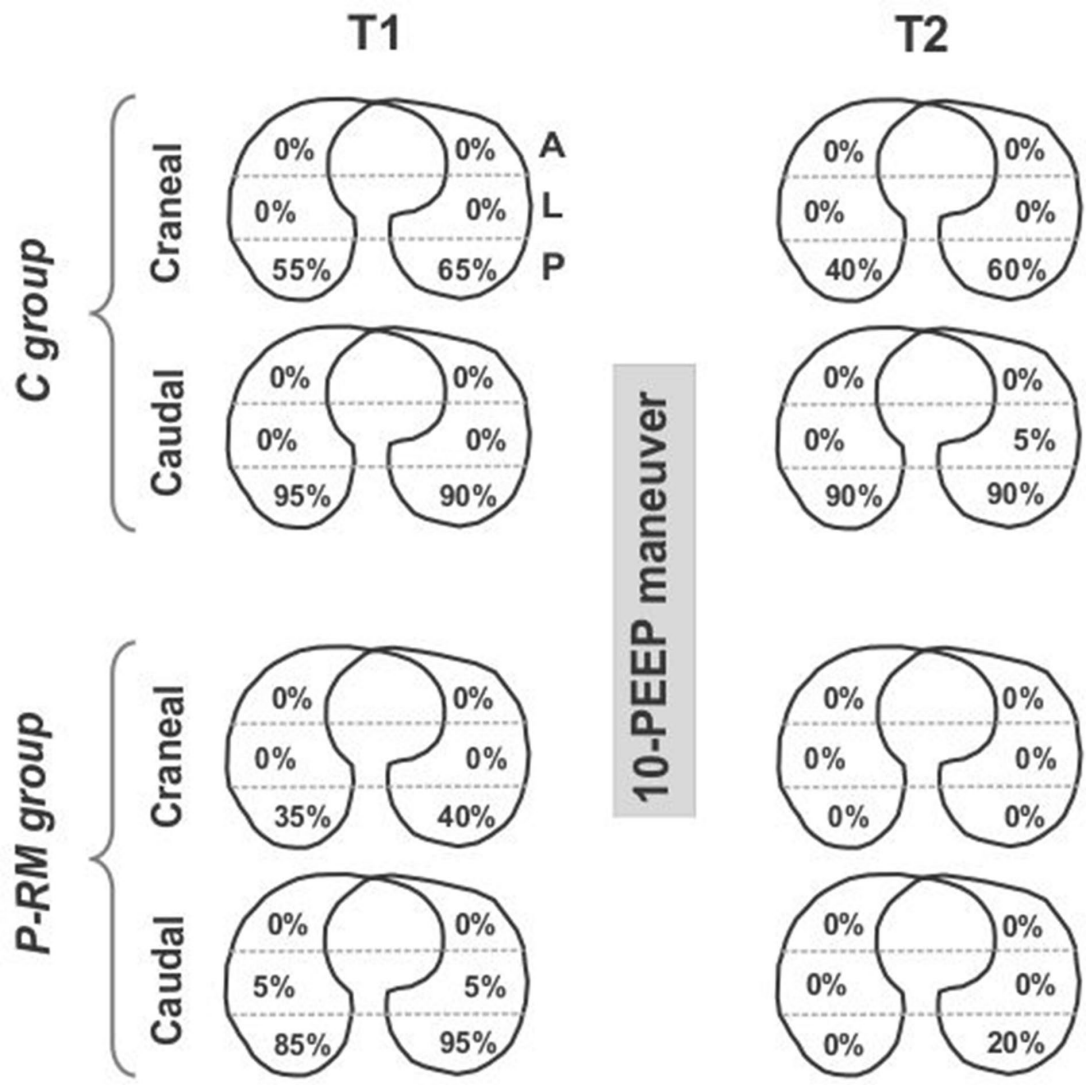
Distribution of atelectasis between groups along the study. *C group* control patients, *P-RM* postural recruitment patients, *A* anterior, *L* lateral, *P* posterior lung zones assessed by lung ultrasound. Cranial = axial cut representing the superior thoracic area above a horizontal line crossing the nipples. Caudal = axial cut representing the inferior thoracic area below a horizontal line crossing the nipples. % = the percent of all patients per group that presented atelectasis in a particular lung zone

Figure 4 shows LUS images of one representative patient per group during the protocol. The distribution of LUS-diagnosed atelectasis is shown in Fig. 5. All patients in the C group presented atelectasis in dependent pulmonary zones at T1 and T2. These lung atelectatic zones were more common in caudal, para-diaphragmatic areas than in cranial areas. After anesthesia induction, the distribution of atelectasis in the P-RM group was similar to the control group. However, most of the atelectasis in patients of P-RM group resolved at the end of surgery (Fig. 5). Only four patients of this latter group showed residual atelectasis after the postural recruitment maneuver.

Table 2 shows hemodynamics and respiratory parameters obtained in both groups. In general, those parameters were statistically similar between groups at each step of the study protocol. PETCO_2_ values were statistically higher at T1 than at T2 in both studied groups. The results of the multiple mixed models are summarized in Table 3. The main finding was that the P-RM caused significant differences in the score of aeration, arterial oxygenation and respiratory compliance. The Air-test, on the other hand, was a good predictor for variations in peak airway pressure.

**Table 3:** Multiple mixed models

Response	Predictive factors				
	Treatment	Age/weight	Gender	Surgery time	Air-test
Score of aeration	< 0.0001	0.477	0.316	0.286	0.264
Heart rate (bpm)	0.298	0.005	0.882	0.441	0.102
MAP (mmHg)	0.940	0.316	0.966	0.241	0.180
SpO_2_ (%)	< 0.0001	0.879	0.013	0.104	0.601
PETCO_2_ (mmHg)	0.407	0.748	0.643	0.794	0.360
Cdyn (mL/cmH_2_O)	0.064	0.104	0.791	0.797	0.428
Pip (cmH_2_O)	0.584	0.454	0.565	0.524	< 0.0001

*MAP* mean arterial blood pressure, *SpO*_*2*_ pulse oximetry hemoglobin saturation, *PETCO*_*2*_ end-tidal partial pressure of carbon dioxide, *Cdyn* dynamic respiratory compliance, *Pip* peak inspiratory pressure

## Discussion

Our study shows that a postural recruitment maneuver can resolve anesthesia-induced atelectasis without the need to reach high airways pressure as during a standard lung recruitment maneuver. A ventilation strategy using the same PEEP and end-inspiratory airways pressure had null effects on atelectasis when patients remained in the supine position. These findings confirm our previous data on the postural recruitment effect [9] and reinforce the feasibility and reproducibility of the P-RM in ventilated pediatric patients with healthy lungs in the clinical field.

Four patients in the P-RM group presented residual atelectasis in the left lower para-diaphragmatic areas (Fig. 5). Previous data showed the high incidence of atelectasis in anesthetized children in this specific lung zone, which could be caused by the pressure that the weight of the heart exerts over the left inferior lobe [4]. Thus, the end-inspiratory airways pressure used during the P-RM and/or the level of PEEP applied after the P-RM were not enough to overcome the alveolar opening and closing pressure, respectively [6]. We hypothesize these patients might need application of higher end-inspiratory airways pressure/PEEP or/and a longer time ventilating in the right lateral posture, to fully resolve this residual lung collapse.

The clinical implication of our results is the chance to treat lung collapse without the need for inducing prolonged high airways pressure. Moderate level of PEEP together with slight increment in end-inspiratory airways pressure for only 3 min was enough to reach complete lung aeration in almost all our patients. The postural change was easy to perform in children. P-RM should be applied when atelectasis appears after anesthesia induction and to resolve any residual atelectasis at the end of surgery. Other potential clinical advantages of P-RM could be the theoretically minor hemodynamics repercussion and less stress on the lung tissue when compared with standard RM performed at higher airways pressure.

Normalizing lung aeration in the perioperative period could be important for patient’s care because: (1) shunt induced by atelectasis is related to hypoxemia, whose incidence in children is high during the perioperative period [1, 16]. (2) Atelectasis constitutes, by definition, a common postoperative pulmonary complication that potentially induces other more severe complications like pneumonia and ventilator-induced lung injury [17–19]. Therefore, resolving atelectasis seems to be a reasonable therapeutic goal in the perioperative period.

### Potential mechanisms explaining the postural recruitment effect

Gravity creates a vertical gradient of *P*_L_ that decreases in the dependent lung areas [20]. Anesthesia-induced atelectasis are mainly caused by a compressive mechanism, where *P*_L_ in the dependent parts of the lungs is low and not enough to offset the compressive forces of thoracic and abdominal content densities, including the weight of the lungs themselves. This vertical gradient of *P*_L_ (*P*_L_ in the superior areas minus *P*_L_ in the bases divided by lung’s height) changes with body mass and posture. Agostoni and D’angelo showed that this gradient ranged from 0.24 cmH_2_O/cm of height in rams to > 0.8 cmH_2_O/cm in rats, which demonstrates that the smaller is the animal the higher is the vertical *P*_L_ gradient [21]. According to this body mass effect demonstrated in animals, it is calculated that adults (similar to mean weight of rams = 75 kg) may potentially have a *P*_L_ gradient close to 0.2 cmH_2_O/cm. Children of different sizes would have a gradient between 0.4 cmH_2_O/cm (similar to weight of small dogs = 15 kg) to 0.6 cmH_2_O/cm (similar to weight of larger dogs = 30 kg) [21]. The same authors showed in rabbits that the *P*_L_ gradient increased when body position was changed from supine (0.55 cmH_2_O/cm) to the lateral position (0.73 cmH_2_O/cm) [22]. These experimental data demonstrate that the lateral position causes higher *P*_L_ in the most ventral lung areas than what could be obtained in the same areas in the supine position, mainly because the thoracic right-to-left distance is longer than the anterior–posterior [9].

The change in body positions during positive pressure mechanical ventilation have been extensively used in pediatric patients with different results [23–26]. The physiological and clinical effects of body positioning on lung function depend on the distribution of perfusion and ventilation. The distribution of ventilation is highly variable in spontaneous breathing children at different positional changes [27]. Conversely, in mechanically ventilated children in lateral position, the distribution of ventilation was more homogeneous between lungs when using PEEP [28]. PEEP improved the ventilation in nondependent lung and increased both, functional residual capacity and PaO_2_. Thus, the lateral position can explain the recruitment effect using normal range of end-inspiratory airways pressure. *P*_L_ becomes larger in the nondependent lung areas due to gravity while PEEP distributes ventilation to the superior lung areas. Then, once recruited, these areas remain “open” with 10 cmH_2_O of PEEP, according to the Laplace’s law, and these nondependent areas become “dependent” during the change in the opposite lateral decubitus.

### Limitations

The P-RM was tested in preliminary patients using different durations and levels of PEEP and end-inspiratory airways pressure. The present study was not designed to analyze the best combination between time and target pressures, but it was ideated just as a proof of concept. In fact, four patients in the P-RM group present some residual atelectasis despite improvement of lung aeration (Fig. 5). The lung opening and closing pressures vary among patients, which means that the P-RM (as well as standard RM) should be personalized to avoid unrealistic and potential harmful higher airways pressure [29]. Next studies should be done to optimize the P-RM settings and its individualization guided by LUS.

LUS is an operator-dependent technique that can induce bias in our results. To avoid inter-observer variability the same investigator performed all scans. Besides, LUS assessment using linear probe of 6–12 MHz in children gives high-resolution images that can diagnose different patterns with high accuracy [13].

We have not analyzed the effect of P-RM after surgery or the repercussion of residual atelectasis in post-operative complications or patient’s outcome. This study was designed to test the feasibility of the postural recruitment concept, and future studies with specific protocols and large populations should be done for these purposes.

## Conclusions

Atelectasis commonly observed in healthy pediatric anesthetized patients is better resolved by a P-RM than by an isolated increase in PEEP. In our study, an increase in PEEP at constant driving pressure maintained for a short time was enough to open up the lungs and keep them open only when combined with sequential changes in body posture. This study confirms the feasibility and efficacy of a P-RM in children with anesthesia-induced atelectasis.

## Data Availability

The data sets are available from the corresponding author on reasonable request.

## Abbreviations

ASA: American Physical Status Classification
Cdyn: Dynamic respiratory compliance
LUS: Lung ultrasound
MAP: Mean arterial blood pressure
PEEP: Positive end-expiratory pressure
PETCO_2_: End-tidal partial pressure of carbon dioxide
Pip: Peak inspiratory pressure
*P*_L_: Trans-pulmonary pressure
Pplat: Plateau pressure
P-RM: Postural lung recruitment maneuver
RM: Lung recruitment maneuver
Rrs: Respiratory airways resistance
SpO_2_: Pulse oximetry hemoglobin saturation
VT: Tidal volume
